# Epididymo-Orchitis as a Presenting Feature of Relapsing Polychondritis: A Case Report

**DOI:** 10.1155/2011/726038

**Published:** 2012-01-04

**Authors:** Kuljeet Bhamra, Rachel Weerasinghe, Alan Steuer

**Affiliations:** Department of Rheumatology, Heatherwood and Wexham Park Hospitals NHS Foundation Trust, Slough, Berkshire SL2 4HL, UK

## Abstract

Relapsing polychondritis (RP) is a rare multisystem disease. It is characterized by recurrent inflammation of cartilaginous structures including the ears, nose, tracheo-bronchial tree and peripheral joints. Proteoglycan-rich structures such as the heart, eyes and blood vessels can also be affected. Systemic symptoms including fever, weight loss and lethargy are common. RP is difficult to diagnose as it presents in a wide variety of ways and there is no diagnostic test. Corticosteroids are the mainstay of treatment but other immunosuppressive drugs can be used in combination with steroids. We present an unusual presentation of RP.

## 1. Introduction

A 74-year-old gentleman presented to the rheumatology clinic with a long and complicated history. His presentation began 6 months previously with an acute episode of bilateral epididymo-orchitis with swollen, red, and painful testes. A presumptive diagnosis of an infectious orchitis was made and he was treated with oral antibiotics. The acute episode eventually settled but he continued to get further episodes of testicular pain and inflammation which generally settled spontaneously. Subsequently, he developed recurrent pleurisy and pericarditis, recurrent inflammation of the left pinna, episodes of conjunctivitis, and inflammation of the nasal bridge. Other symptoms included intermittent musculoskeletal chest wall pain and atrial tachyarrhythmias. He developed an erythematous rash over both legs. During this 6 month period, he suffered dramatic weight loss.

Clinical examination confirmed an inflamed left ear with sparing of the lobe, his nasal septum was tender, and there was a vasculitic rash over both legs.

Investigations revealed a normocytic anaemia (Hb 95 g/L MCV 95fl), CRP 106 mg/L, ESR 128 mm/hr, serological testing for the following were negative: ANA, ENA, RF, and ANCA. Echocardiogram confirmed mild aortic regurgitation. Ultrasound scan of the testes revealed hydroceles and enlarged epididymises bilaterally. Urinanalysis was normal.

**Figure 1:**
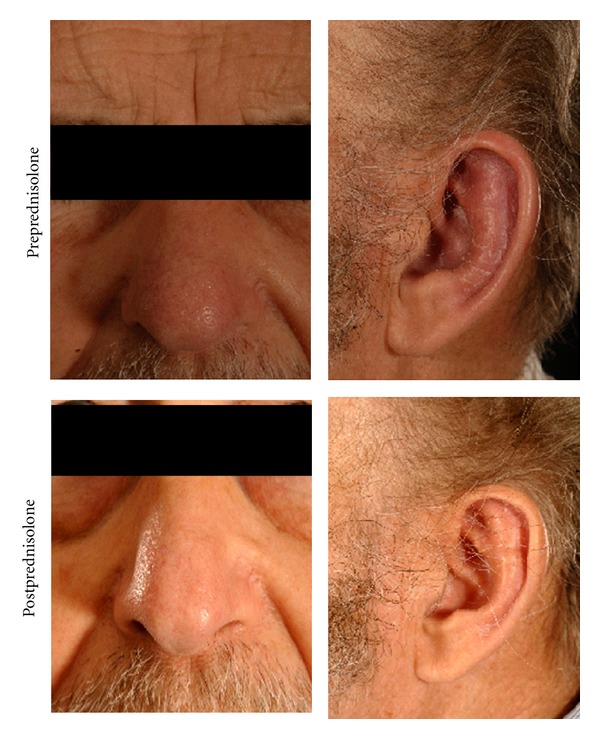
Erythema of nasal and auricular cartilage with ear lobe sparing pre and post treatment with prednisolone.

Applying the McAdams criteria for diagnosis [1].  RP was diagnosed based on multiple areas of chondral inflammation including the ear, nose, eyes, and chest wall chondritis, He was admitted to hospital and started on high dose oral prednisolone (Figure 1). Within 3 days of starting steroids, there was a dramatic improvement in his symptoms with complete resolution of chest pain, ear, and nasal inflammation and conjunctivitis. Subsequent bone marrow aspiration confirmed myelodysplasia. Over the subsequent 12 months, the patient became transfusion dependent. He had no further episodes of epididymo-orchitis.

## 2. Discussion

RP is a rare multisystem disease that in one study had an estimated annual incidence of 3.5/million [2].  RP is characterized by recurrent episodes of inflammation of cartilaginous structures including the ears, nose, tracheobronchial tree, and peripheral joints.

The aetiology of RP is unknown. Evidence suggests the disease is produced by an immunological process. Antibodies to type-II collagen have been identified in the sera of patients with RP and were first identified during acute attacks and seemed to correlate with disease severity [3]. Moreover, an experimental animal model was generated by sensitizing rats with type II collagen [4, 5]. More recently, a link has been made with the HLA-DR4 antigen [6]. 

This case is unusual as the first presenting symptom was epididymo-orchitis. Orchitis was first described in association with RP in 1994 [7].  RP usually presents with ear, nasal, or throat pain but initial symptoms do tend to vary which is why there is often a delay in diagnosis with an average of 2.9 years to diagnosis [8].  RP must be diagnosed on clinical grounds as there is no diagnostic test. Vasculitis is (the most common) an associated feature and (as shown by this case) can involve the vessels of the testes [1]. Aortic regurgitation is likely to be due to dilatation of the aortic ring caused by vasculitis [9]. 

Myelodysplastic syndromes are a group of neoplastic disorders of the bone marrow resulting in abnormalities of the myeloid cell lineage. An association with RP has been reported, but the underlying mechanism of this association remains obscure and difficult to prove; possibly due to the rarity of RP [10, 11]. In 1986, Michet et al. reviewed 112 cases of RP, 6 of whom developed haematological malignancies in the form of myeloproliferative or myelodysplastic conditions, identifying anaemia as a poor prognostic marker [9, 10], as found in our patient who despite immunosuppression therapy had a persistent anaemia eventually becoming transfusion dependent.

## 3. Conclusion

Testicular and epidydmal inflammation must be included on the list of presenting features of RP.
